# Haplotype analysis of the 5,10-methylenetetrahydrofolate reductase (MTHFR) c.1298A>C (E429A) polymorphism

**DOI:** 10.1186/1756-0500-4-439

**Published:** 2011-10-24

**Authors:** Alexander Semmler, Susanna Moskau, Holger Lutz, Peter Meyer, Michael Linnebank

**Affiliations:** 1 Department of Neurology, University Zurich, Frauenklinikstrasse 26, 8091 Zurich, Switzerland; 2 Department of Neurology, University Bonn, Sigmund-Freud-Strasse 25, 53105 Bonn, Germany; 3 Institute of Molecular Medicine, Sperberstr. 2, 81827 Munich, Germany

## Abstract

**Background:**

The polymorphism 5,10-methylenetetrahydrofolate reductase (MTHFR) c.1298A>C is associated with various diseases. 45 DNA samples homozygous for the A allele and 40 DNA probes homozygous for the C allele were taken from healthy German subjects of white Caucasian origin to analyze the haplotype of the two MTHFR c.1298A>C alleles. Samples were genotyped for the polymorphism MTHFR c.677C>T and for the silent polymorphisms MTHFR c.129C>T, IVS2 533 G>A, c.1068C>T and IVS10 262C>G.

**Findings:**

Haplotype construction revealed that the C-allele of MTHFR c.1298A>C was more frequently observed *in cis *with c.129T, IVS2 533A, c.677C, c.1068T, and IVS10 262 G than expected from normal distribution. Estimation of the most recent common ancestor with the DMLE + 2.3 program resulted in an estimated age of approximately 36,660 years of the MTHFR c.1298C allele.

**Conclusion:**

Given that the era from 30,000 to 40,000 years ago is characterised by the spread of modern humans in Europe and that the prevalence of the MTHFR c.1298C allele is significantly higher in Central Europe in comparison to African populations, a selective advantage of MTHFR c.1298C could be assumed, e. g. by adaption to changes in the nutritional environment. The known founder ancestry of the T allele of MTHFR c.677C>T allele, together with the present data suggests that the MTHFR mutant alleles c.677T and 1298C arose from two independent ancestral alleles, that both confer a selective advantage.

## Background

The monomeric enzyme 5,10-methylenetetrahydrofolate reductase (MTHFR, EC 607093, OMIM 236250) catalyzes the reduction of 5,10-methylenetetrahydrofolate to 5-methyltetrahydrofolate (5-MTHF). 5-MTHF is a methyl group donor for the remethylation of homocysteine to methionine. The T-allele of the MTHFR polymorphism c.677C>T (A222V, rs1801133) is associated with reduced MTHFR activity and, thus with an increased total plasma homocysteine level [1]. The frequency of the T-allele of MTHFR c.677C>T differs between ethnic groups and ranges from 6-10% in African countries [2,3] to more than 17% in Caucasians in North America [1,4,5] and more than 50% in Mexican populations [3]. In the literature, the presence of the T-allele has been associated with cardiovascular and cerebrovascular diseases, venous thrombosis, neural tube defects and various cancers [6]. It was hypothesized that this might be due to increased plasma homocysteine levels in carriers of the MTHFR c.677T allele [7]. In addition, the allele's function for DNA synthesis and methylation could be an important factor for the development of such diseases [8]. Previously, we demonstrated that the polymorphism MTHFR c.677C>T (A222V, rs1801133) is associated with a defined MTHFR haplotype in the German population suggesting a founder effect [9]. This result could be confirmed by other groups for other populations [10].

In the present study, we investigated the second functionally relevant MTHFR SNP, the c.1298A>C (E429A, rs1801131) [11]. The derived C-allele of MTHFR c.1298A>C has been found to be associated with decreased MTHFR activity in lymphocytes and cultured human fibroblasts, although this effect was smaller than the effect shown for the T-allele of MTHFR c.677C>T [11-13]. However, the C-allele of the MTHFR c.1298A>C polymorphism could be linked to later onset of neurodegenerative diseases and a lower risk for different types of cancer [12,14]; [15-18]. Here, we report haplotype data on the c.1298A>C polymorphism in a healthy German population selected by the c.1298A>C genotype suggesting a founder allele in the late ancestry.

## Methods

The MTHFR c.1298A>C polymorphism (rs 1801131) was studied in samples of genomic DNA prepared from peripheral leukocytes of 500 healthy controls recruited for previous studies [19,20]. All individuals were Caucasians from Germany living in the area of Bonn. The distribution of AA/AC/CC was 260/200/40 or 0.52/0.40/0.08, respectively, which was in line with the Hardy-Weinberg-Equilibrium. Out of 500 DNA samples, we selected 40 samples that were homozygous for the C allele and 45 samples demonstrating homozygosity for the A-allele. All samples were genotyped for the missense polymorphism MTHFR c.677C>T (rs 1801133) and for the silent polymorphisms MTHFR c.129C>T (rs 2066462), IVS2 533 G>A (polymorphism at nucleotide number 533 in intron 2 [21]), c.1068C>T (rs 2066462) and IVS10 262C>G (polymorphism at nucleotide number 262 in intron 10 [21,22]. Haplotypes were constructed from genotypes using the maximum likelihood method (Software HAPMAX, developed by M. Krawczak, http://www.uni-kiel.de/medinfo/mitarbeiter/krawczak/download/) and analysed with a χ^2 ^test (one degree of freedom). Estimation of the most recent common ancestor of the derived allele of MTHFR c.1298C was performed by means of the DMLE + 2.3 program http://www.dmle.org[23]. The chromosome map distance of the polymorphisms was calculated from the pubmed SNP database http://www.ncbi.nlm.nih.gov/SNP/snp_ref.cgi?locusId=4524 (Table 1). The population growth rate in Germany was set to 0.0075 [24], the current German population to 82,200,000 (Population Reference Bureau. 2008 World Population Data Sheet) and the mean allele frequency of the MTHFR c.1298C allele to 28% according to the results from the present study derived from 500 samples which was in line with results from other German populations [14,20,25,26]. The generation length was defined as 20 years.

**Table 1:** Chromosome map distances

SNP	Contig position NT_021937	Distance in bp to c.129C>T
c.129C>T	6.400.338	0

IVS2 533 G>A	6.398.006	2.332

c.677C>T	6.393.745	6.593

c.1068C>T	6.392.263	8.076

c.1298A>C	6.391.843	8.495

IVS10 262C>G	6.388.372	11.966

The study was approved by the local ethical committee of the University Bonn, Germany, and all individuals gave written informed consent.

## Results

**Figure 1:**
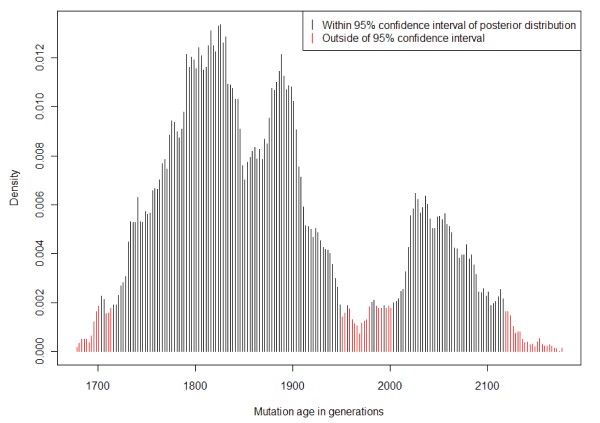
**Estimation of the most recent common ancestor of MTHFR c.1298C with the DMLE + 2.3 program**.

Selection of DNA samples was based on their MTHFR c.1298A>C genotype. Therefore, we only performed haplotype analysis stratified for this genotype and not for the other MTHFR SNPs since representative distribution of these SNPs in our sample could not be assumed. HAPMAX analysis revealed a linkage disequilibrium of MTHFR c.1298A>C to the other SNPs. The C-allele of MTHFR c.1298A>C was more often observed *in cis *with c.129T; IVS2 533A; c.677C; c.1068T; IVS10 262 G than expected from a normal distribution referring to the haplotype data obtained for the wildtype A-allele (Table 2). Accordingly, automated haplotype construction revealed different haplotypes of maximum likelihood for c.1298A and c.1298C (Table 3). Estimation of the most recent common ancestor of the MTHFR c.1298A>C alleles with the help of the DMLE + 2.3 software showed a major peak of the posterior distribution of 1833 generations (95% credible set, 1678-2176 generations) (Figure 1). Given a generation length of 20 years, this finding translates in an age of approximately 36,600 years (33560-435200).

**Table 2:** Relative frequency of polymorphisms of A- and C-alleles of MTHFR c.1298A>C, Pearson's Chi-Square Test.

	c.129C>T			IVS2 533 G>A			c.677C>T			c.1068C>T			IVS10 262C>G		
	**C**	**T**	**Chi^2^;** **p**	**G**	**A**	**Chi^2^;** **p**	**C**	**T**	**Chi^2^;** **p**	**C**	**T**	**Chi^2^;** **p**	**C**	**G**	**Chi^2^;** **p**

1298 A	0.99	0.01	15.92	0.84	0.16	19.81	0.49	0.51	52.63	0.99	0.01	15.92	0.70	0.30	30.53

1298 C	0.81	0.19	< 0.001	0.52	0.48	< 0,001	0.99	0.01	< 0.001	0.81	0.19	< 0.001	0.28	0.72	< 0,01

**Table 3:** Haplotypes of A- and C-alleles of MTHFR c.1298A>C.

HT	c.129C>T	IVS2 533 G>A	c.677C>T	c.1068C>T	IVS10 262C>G	1298A	1298C
1	C	G	C	C	C	0.3201	0.1043

2	C	G	T	C	C	0.2956	0

3	C	G	T	C	G	0.1113	0

4	C	G	C	C	G	0.1026	0.2287

5	C	A	T	C	C	0.0661	0

6	C	A	C	C	G	0.0432	0.3468

7	C	A	T	C	G	0.0384	0

8	T	A	C	C	C	0.0114	0.1279

9	C	G	C	T	C	0.0114	0.1128

10	T	G	C	T	C	0	0.0390

11	T	A	C	T	C	0	0.0160

12	T	G	C	T	C	0	0.0122

13	C	G	T	T	C	0	0.0122

Haplotypes with an estimated frequency below 0.01 are not shown. Numbering refers to haplotypes of c.1298A alleles (1-13; the additional c.1298 haplotypes not observed in c.1298A (C) alleles were numbered consecutively regarding their frequency). Frequencies are shown as the ratio of total amount of each 1298A and 1298C-alleles.

## Discussion

In our German population the A- and the C-allele of MTHFR c.1298A>C demonstrated different haplotypes. The most recent common ancestor of these alleles was calculated with approximately 36,600 years. However, analysis of additional populations is required to definitely determine the age of the ancestor allele.

The selection of samples for homozygosity was performed to achieve an easier allocation of haplotypes to the A versus C allele which would have been more difficult in heterozygotes (AC) for MTHFR c.1298A>C. Due to this biased selection, the observed frequencies of the other MTHFR SNPs are not representative for a general population. Nevertheless, this approach was feasible to approximate haplotype frequencies in A versus C alleles of MTHFR c.1298A>C and to estimate the age of the derived allele, c.1298C. Although the DMLE program predicted the most likely age to be approximately 36,600 years according to the major peak, our analysis revealed at least one additional lower peak (Figure 1) that might be artificial or due to the presence of an undefined subpopulation in our study cohort. This as well as the large confidence interval of the estimated age requires replication of our findings in additional samples and other populations.

Our age estimation of the most recent common ancestor of the studied alleles falls into a time period (30,000 to 40,000 years ago, the Aurignacian) which is typically associated with the spread of modern humans (Homo sapiens) in Europe [27,28]. The frequency of the MTHFR c.1298C allele is significantly higher in central Europe in comparison to African populations [3]. In addition, dietary folate intake can profoundly modify the effect of the C-allele on disease association, and the availability and intake of folate is different in Europe and Africa [29]. In summary, this could indicate an increased frequency of the C-allele due to changes in the (nutritional) environment leading to selective advantages of the C-allele.

Presence of the C-allele has been associated with a later onset of neurodegenerative diseases [12,14], better fertility (however, only observed for *in vitro *fertilisation) [30], rarer occurrence of chromosomal aberrations (Down's Syndrome [31]), and lower cancer risk [15-18]. However, adverse effects like an increased risk of neural tube defects [11], hypertension [32] or acute myeloid leukemia in Brazilian children [33] were also described.

Besides historical genetic aspects, the haplotypes presented in this study might be helpful for perinatal diagnosis of MTHFR deficiency based on haplotype analysis in the case of an unknown MTHFR mutation (OMIM 236250). Furthermore, haplotypes might be used for loss of heterozygosity studies comprising chromosome 1p36, which is involved e.g. in glioblastoma formation [34]. In addition, the known linkage disequilibrium of the two MTHFR c.677T and c.1298C alleles [35,36] was reconfirmed in our study.

## Conclusion

The previously described founder effect for the c.677T allele [9] supported by the present data suggests that the MTHFR c.677T and c.1298C alleles arose from two independent ancestral alleles, that both confer a selective advantage.

## Competing interests

The authors declare that they have no competing interests.

## Authors' contributions

AS participated in the study design and genetic studies, carried out the statistical analysis and drafted the manuscript, HL and SM participated in the genetic studies, PM participated in drafting the manuscript, ML participated in the study design, statistical analysis and drafting of the manuscript. All authors read and approved the manuscript.
